# The role of access to finance from different finance providers in production risks of horticulture in Indonesia

**DOI:** 10.1371/journal.pone.0257812

**Published:** 2021-09-27

**Authors:** Eliana Wulandari, Miranda P. M. Meuwissen, Maman H. Karmana, Alfons G. J. M. Oude Lansink

**Affiliations:** 1 Faculty of Agriculture, Universitas Padjadjaran, Jatinangor, West Java, Indonesia; 2 Business Economics, Wageningen University, Wageningen, The Netherlands; Szechenyi Istvan University: Szechenyi Istvan Egyetem, HUNGARY

## Abstract

This paper aims to investigate the association between perceived production risk of horticultural farms and access to finance from different finance providers. The collection of data was conducted among 434 farmers in Indonesia particularly farmers produced mango, mangosteen, chili and red onion. Production risk was measured by the coefficient of variation, skewness, kurtosis and the 25^th^ percentile. Depending on the risk measure, a lower production risk was found for commercial credit from banks and flexible payments of inputs to kiosks. Conversely, we did not find any lower production risk associated with finance provided by farmers’ associations and other sources such as family and friends. Results of this study are useful for policy makers, finance providers and supply chain actors in agriculture. For policy makers, the results of this study can help in designing policy to enhance farmers’ access to finance, whereas finance providers and supply chain actors, such as traders, obtain insight into whether their financial provisions effectively reduce production risk.

## Introduction

Production risk is an important issue for policy makers, finance providers and supply chain actors in agriculture, such as farmers and traders. Production risk is important especially in developing countries. Risk exists in agricultural production because of the high variability of production that cannot be certainly predicted by farmers [1]. Previous studies have shown that production risk has a large impact on the decision to invest in farm technology [2], which in turn affects food production and therefore food security [3, 4].

Production risk, among other reasons, has led to a decline in the number of farm households, which could affect agricultural production in Indonesia [5], and therefore food security in Indonesia [6]. Factors affecting production risk include climate factors, such as extreme rainfall [7–9], pests and diseases, labor, and the quality of seeds [8].

With regard to the climate factors, high density of rainfall and drought in several places in Indonesia has led to crop failure [10], as illustrated by excessive rainfall causing floods and subsequent crop failures for farmers [11], and droughts leading to drastic decline of crop yields [12]. Besides climate factors, pest and disease have also affected agricultural production in Indonesia. Several studies reported that farmers faced production risk because of monkeys and wild pigs [13] and leaf miner attacks [14].

Studies have shown that production risks can be minimized by several practices, for instance by applying high quality seeds [15, 16], pesticides [17] and [18] fertilizer. Furthermore, production risk can be reduced by undertaking activities that increase yields; these activities can be facilitated by proper access to finance [19, 20]. For instance, farmers who have access to credit can adopt intensive agriculture [21] and can invest in irrigated land, which can reduce the effects of climate variability and therefore reduce production risk [20]. Credit can be used to purchase inputs that can decrease production risk, such as feeds, fertilizer, insecticides and fungicides [19]. Therefore, finance may play an important role in conducting such practices.

Finance can be accessed from different types of finance providers; these providers generally facilitate access to different agricultural inputs. For example, credit can be used to buy agricultural inputs and improved technologies [22]. Credit can be obtained from a variety of sources including banks, moneylenders, relatives, cooperatives [23, 24], micro finance institutions (MFIs) [25, 26] and various development programs [24]. Farmers can also obtain finance through government subsidies. Subsidies, especially in developing countries, are provided for agricultural inputs, such as fertilizer and seeds, and for interest payments on credit [27, 28]. In-kind finance, such as fertilizer and seeds, has been provided by a village association [29] and traders [30, 31]. A study of Wulandari et al. [28] reported that Indonesian farmers can access finance from many different sources, such as credit from banks and MFIs. While most banks and MFIs provide commercial credit, some banks also provide a subsidized credit program, for instance the program of micro credit loans (*KUR*-*Kredit Usaha Rakyat*). This is offered by banks collaborating with the Indonesian government. The authors also found that farmers can access in-kind finance, offered by farmers’ associations and traders. In addition, agricultural input kiosks offer the opportunity for flexible payments for inputs. Besides, farmers were found to have access to finance from other sources such as family, relatives and neighbors.

Despite the importance of access to finance in reducing production risk, to the best of the authors’ knowledge, no studies have analyzed the effect on production risk of different sources of finance. In this context, this study aims to analyze the effect of finance from different finance providers on production risk in Indonesia. Finance providers included in the study are banks, MFIs, farmers’ associations, traders, agricultural input kiosks and other finance sources. Results of the study provide insight into the role of different finance sources in minimizing production risk, especially in developing countries such as Indonesia. This information is useful for policy makers, finance providers and supply chain actors in agriculture. For policy makers, the results of this study can help in designing policy to enhance farmers’ access to finance; whereas finance providers and supply chain actors, such as traders, obtain insight into whether their financial provisions effectively reduce production risk. This study focuses on horticultural farmers because horticulture is important for agricultural GDP and the growth of employment in agriculture in Indonesia [32].

This paper proceeds with a description of finance sources available to farmers in Indonesia. This is followed by a description of the methods and variables used. Section 4 presents the results for the measures of production risk and the effects of the explanatory variables on these measures. Finally, the paper discusses the results and provides policy implications.

## Materials and methods

### Questionnaire design

To measure production risk and access to finance, structured questionnaires for farmers were prepared and pre-tested to evaluate consistency and clarity, and to avoid duplicate questions. Pretesting questionnaires ensure respondents can easily understand questions that being asked and enable to answer the questions. Collecting data from respondents using a questionnaire is the best way in a sampling survey [33]. The questions covered two main areas: an assessment of perceived yields and previous access to finance from different sources.

### Data collection

The survey was conducted from January to July 2014. Data were collected in person from farmers who cultivate one of the four selected crops as their main output. The selected crops were mango, mangosteen, chili and red onion; these crops are identified by the Agricultural Ministry of Indonesia as key crops for horticultural development in Indonesia [34]. The 434 farmers who were surveyed were selected from the main areas of horticultural production, i.e. West Java, Central Java and East Java. The selection procedure is explained in the following paragraphs.

Within Java, study sites were selected based on the importance of horticultural production, especially for the selected crops. Java is divided into provinces. Firstly, two provinces were selected as study sites for each selected crop, based on the largest production area and the potential to develop the selected crop. According to data from the Central Bureau of Statistics in Indonesia [35], production of mango occurs mainly in West Java and East Java, and the production of chili, red onion and mangosteen occurs mainly in West Java and Central Java.

Next, for each province, districts with the highest production during the last five years were selected. For mango, the chosen districts were Cirebon and Indramayu (West Java), and Probolinggo and Pasuruan (East Java). The chosen districts for mangosteen were Tasikmalaya and Subang (West Java), and Purworejo (Central Java). For chili, selected districts were Garut, Tasikmalaya and Ciamis (West Java), and Pemalang and Purbalingga (Central Java). Finally, the selected districts for red onion were Majalengka and Bandung (West Java), and Brebes (Central Java).

Farmers were then randomly selected in each district, based on farm address data obtained from agricultural officers and personal contacts. Our sample contained 434 farmers who were grouped according to their main crop: 101 producing mango, 103 producing mangosteen, 123 producing chili and 107 producing red onion.

As a part of a comprehensive project, this study was reviewed and approved by the Assessment Committee of Wageningen School of Social Sciences (WASS). We used supporting letters of this study from the project team, accompanied with a letter from Indonesian embassy in The Netherlands and a letter from Faculty of Agriculture, Universitas Padjadjaran, Indonesia, in which these letters were used during the survey of farmers. We obtained permission from the agricultural offices in study areas. Prior to the interviews, oral consent was obtained from the farmers because part of the target participants had little schooling. A previous study of Reyes-Garcia et al. [36] had a similar procedure. We introduced the farmers to the purpose and contents of this study before entering the survey. The participation of farmers was voluntary and they were assured that anonymity would be treated with an anonymous data set.

### Elicitation of yields

Historical data on farm yields was unavailable for individual farmers. We therefore chose to elicit perceived yields, an approach also used by Ghadim & Pannell [37]. First, we asked the farmers about the lowest and highest perceived yields that could be obtained in the next five years, given the current use of inputs, access to finance, disease pressure and physical growth conditions. Yields were specified in kilograms per hectare. Next, the range of the perceived yields was divided equally into seven intervals. Then, 20 coins were given to the farmers, with each coin representing a 0.05 probability. Farmers were asked to distribute all the coins over the seven intervals, in accordance with their expectation of the likelihood of obtaining the yield represented by each interval. All the farmers were able to perform the elicitation task assisted by an interviewer. The descriptive statistics of the perceived yields are presented in Table A, in S1 Appendix.

### Measurement of production risk

To measure production risk, we used four measures of risk derived from the elicited yield data: the coefficient of variation (CV), skewness, kurtosis and the 25^th^ percentile. The first three measure the shape of the yield distribution, whereas the 25^th^ percentile measures an absolute value of the distribution. The CV was chosen to represent production risk because it measures the variability of the data. Coefficient of variation has been widely used to measure variation in many applied disciplines including finance and engineering, and has facilitates to compare variability of two or more groups [38]. CV was used to measure risk in terms of how much the data are spread out (variation) [39].

Risk was also measured by kurtosis and skewness. Skewness measures the degree of asymmetry of the distribution particularly in how much the distribution is skewed to the left or right [40]. As explained by Kobayashi et al. [41] skewness has values that are symmetrically distributed around zero, that is negative skewness is occurs when the left tail of the distribution is longer than the right tail, while positive skewness occurs when the right tail of the data is longer than the left tail. Kurtosis measures the nature of distribution tails particularly in terms of their length and weight [40]. Furthermore, Vose [42] explained that kurtosis measures the peak of a distribution compared to the normal distribution, which has a kurtosis of three. A higher kurtosis indicates that the distribution becomes narrower, which indicates lower production risk.

The last measure of risk used in this paper was the 25^th^ percentile, which measures the absolute value of the risk. The 25^th^ percentile of the production is a measure of the downside risk of production. The 25^th^ percentile applied in this paper is the first quartile representing the lowest amount of resources available Melnyk et al. [43]. The 25^th^ percentiles of perceived yields were rescaled between 0 and 1 by subtracting the minimum value from each yield value and dividing the result by the difference between the maximum and minimum values. The higher the 25^th^ percentile, the lower the production risk in terms of the yield level.

### Factors explaining production risk

In the next stage of the analysis, the scores for the production risk measures were regressed on finance and socio-economic factors. Ordinary least square (OLS) was used to determine which factors are associated with the production risk measures. The generic form of the OLS model is:

$${\text{y}}_{\text{i}}={\text{x}}_{\text{i}}\;\beta +\varepsilon_{\text{i}},\;\text{i}=1,2,\ldots \ldots ,\text{N}\left(434\;\text{farmers}\right)$$ (1)

where the dependent variable y_i_ is the risk measure of perceived yields. The independent variables (x_i_) were finance factors representing access to different sources of finance, socio-economic factors and a variable representing farm specialization.

The finance factors are dummy variables indicating whether the farmers had access to finance from these finance providers. Seven finance providers were included: commercial (*commercial credit*) or subsidized credit (*subsidized credit*) from a bank, commercial credit from a MFI (*mfi*), in-kind finance from a farmers’ association (*farmers’ association*) or trader (*trader*), flexible payment of inputs to an agricultural input kiosk (*kiosk*), or finance from other sources of finance (*other*) such as from family, relatives and friends. In case a farmer had multiple access to finance from different sources, we counted for each access. For instance, if a farmer had access to finance from a farmers’ association, agricultural input kiosk and a friend, then we counted the dummy of one for access to finance to each of these finance sources i.e. to *farmers’ association*, *kiosk* and *other*.

The model also included socio-economic factors: age, education, farming experience, farm size, distance to agricultural input kiosk and infrastructure of roads. Age (*age*) is measured as the age of the farmer (years), education (*education*) is the number of years of formal education of the farmer, and farming experience (*fexp*) is farmer experience in managing their farms (years). Farm size (*fsize*) is measured as the size of area used by the farmers for producing crops. Infrastructure of roads (*infrastructure*) is a dummy variable, with 1 indicating good roads, and distance to agricultural input kiosk (*distance*) is measured as the distance from the farmer’s house to the nearest agricultural input kiosk (kilometers). The farm specialization variable is a dummy variable, for which mango is the reference crop.

The finance variables cover all the sources of finance available to horticultural farmers in Indonesia. Finance from all these sources is expected to reduce production risk on farms because it facilitates access to inputs and technologies. Commercial credit from banks is often for larger loans, which can be used to improve irrigation to boost production [20], which may lead to more robust yields and hence reduce production risk. Subsidized credit from banks is granted mostly for investing in agricultural equipment or renovating old buildings [44], which may help farmers to reduce the riskiness of production. MFIs provide necessary financial means to obtain both higher quantities and better quality of inputs [22]; credit from MFIs may thus also help to reduce production risk.

With regard to in-kind finance, the provision of inputs to association members [29] and to farmers with a contract with traders [30], and the provision of flexible payments for inputs from an agricultural input kiosk all facilitate the access to agricultural inputs, which could decrease a production risk. Farmers need to buy agricultural inputs during the planting and growing periods, while they earn money after crops are harvested [45]. Bozoglu & Ceyhan [46] showed that the majority of farmers have a problem of negative cash flow during planting and growing periods. Finance from informal sources, such as family, relatives and neighbors help farmers to purchase agricultural inputs during planting and growing periods [47], which could reduce production risk.

Socio-economic variables that were expected to affect production risk were chosen based on literature. Age is expected to reduce production risk because older farmers focus on improving production, especially after investment and expansion [48]. Education is expected to reduce production risk because more educated farmers use improved technologies more productively on their farms [49, 50] and have a better knowledge on the aspect of production, marketing and business [51]. Furthermore, more farming experience and larger farm size are also expected to reduce production risk. For instance, Gebrehiwot & van der Veen [52] found that more experience and larger farms increased the likelihood of adapting to climate change by using crop diversification, which may lead to reduce production risk. The distance from the farmer’s house to the agricultural input kiosk and infrastructure of roads are both expected to reduce production risk. Close proximity to the kiosk is expected to reduce production risk because access to input markets is very effective in increasing production [53]. Similarly, good roads are expected to reduce production risk by increasing access to services, such as agricultural input markets [54] and the credit market [55].

Before carrying out the regression, all the independent variables, except for the dummy variables, were standardized to prevent scale effects of the β variables. We tested for homoscedasticity using the Breusch-Pagan test and checked for multicollinearity by calculating the variance inflation factors (VIF) for each variable. Following Rook et al. [56], we did not find any multicollinearity in the model, as all VIF values were below 10. Following Melnyk et al. [43], the robust regression was applied in the model with regard to normality and outliers. With regard to reverse causality, we believe that potential reverse causality of production risks affecting the success of obtaining finance from different finance providers does not play a role in our model. To the best of authors’ knowledge, there is no local evidence or literature indicating that finance providers in Indonesia require farmers to provide information related to production risks as part of the loan assessment procedure. For instance, to minimize risk, banks in general require collateral [57] and check the loan redemption track record of credit applicants through the Bank of Indonesia [58]. To access finance from the Indonesian government, the sole requirement for farmers is to submit an agricultural business plan through a farmers’ association [28].

The characteristics of farmers are presented in Table 1. The table shows that, in terms of the finance factors, the majority of the farmers (28%) had access to finance from other financial providers, such as family, relatives and neighbors. With regard to financial access from different sources of finance, a large percentage of the mango farmers (45%) obtained finance from other sources, such as family, relatives and neighbors. In-kind finance from farmers’ associations was obtained by 39% of the mangosteen famers and 41% of the chili farmers obtained finance from traders. A large group of red onion famers obtained finance from agricultural input kiosks (42%). Regarding the socio-economic factors, farmers were, on average, 47 years old with eight years of formal education, in which chili farmers were the youngest farmers. In addition, the farmers had an average of 24 years of farming experience.

**Table 1:** The characteristics of farmers.

Variables		Mango		Mangosteen		Chili		Red Onion		Overall	
		(n = 101)		(n = 103)		(n = 123)		(n = 107)		(n = 434)	
		Mean	SE	Mean	SE	Mean	SE	Mean	SE	Mean	SE
Finance factors*											
**Bank**	**Commercial credit**	0.18	0.04	0.13	0.03	0.11	0.03	0.05	0.03	0.11	0.02
	**Subsidized credit**	0.06	0.02	-	-	0.35	0.04	-	-	0.11	0.02
**MFI**		0.04	0.02	0.11	0.03	0.02	0.01	0.08	0.03	0.06	0.01
**Farmers’ association**		0.25	0.04	0.39	0.05	0.15	0.03	0.23	0.04	0.25	0.02
**Trader**		0.09	0.03	0.05	0.02	0.41	0.04	0.07	0.02	0.17	0.02
**Kiosk**		0.08	0.03	0.01	0.01	0.20	0.04	0.42	0.05	0.18	0.02
**Other**		0.45	0.05	0.14	0.03	0.30	0.04	0.23	0.04	0.28	0.02
**Socio-economic factors**											
**Age**		46	0.93	53	1.11	41	0.86	49	0.91	47	0.52
**Education**		9	0.25	8	0.26	8	0.25	7	0.22	8	0.13
**Farming experience**		23	1.13	31	1.42	15	0.86	28	1.17	24	0.64
**Farm size**		1.30	0.30	0.87	0.07	1.20	0.20	0.69	0.15	1.02	0.10
**Distance to kiosk**		3.38	0.29	3.82	0.32	2.07	0.17	1.13	0.11	2.56	0.13
**Infrastructure**		0.48	0.05	0.38	0.05	0.25	0.04	0.49	0.05	0.39	0.02

*had access to finance from each finance providers: Commercial or subsidized credit from bank, commercial credit from MFI, in-kind finance from farmers’ association or trader, flexible payment of inputs to agricultural input kiosk, or finance from other sources of finance such as from family, relatives and friends.

Source: Authors’ own calculation.

## Results and discussion

### Production risk of horticultural farms

Table 2 presents the four measures of production risk for the four groups of farms, grouped by main crop. The CV results show that the yield variability was highest for mangosteen farms (0.28), followed by mango (0.25), chili (0.24) and red onion (0.20). Furthermore, the yield skewness was negative for all crops, which suggests a yield distribution with an asymmetric tail extending towards lower yields. The skewness ranged between -0.32 (red onion) and -0.02 (mango). Table 2 also shows that the kurtosis was less than three for all crops, with values between 2.20 (mango) and 2.86 (chili). This indicates a relatively flat yield distribution. Furthermore, Table 2 also shows that the 25^th^ percentiles of perceived yields, which indicate the absolute value of production risk, ranged between 0.21 (mango) and 0.32 (red onion).

**Table 2:** Measures of production risk.

Variables	Mango	Mangosteen	Chili	Red Onion
**Coefficient of variation**	0.25	0.28	0.24	0.20
	(0.01)	(0.02)	(0.01)	(0.01)
**Skewness**	-0.02	-0.08	-0.10	-0.32
	(0.05)	(0.06)	(0.07)	(0.06)
**Kurtosis**	2.20	2.32	2.86	2.51
	(0.06)	(0.12)	(0.22)	(0.11)
**25** ^**th**^ percentile*	0.21	0.27	0.30	0.32
	(0.02)	(0.02)	(0.02)	(0.02)

Mean values with standard errors in parentheses.

* The unit was rescaled on a scale from 0 to 1 by subtracting the minimum value from each yield value and dividing the result by the difference between the maximum and minimum values.

Source: Authors’ own calculation.

### The association between access to finance and socio-economic factors and the production risk of horticultural farms

Table 3 shows the outcomes of the regression of production risk measures on socio-economic factors and the factors representing access to finance. Results show that the association between risk and access to finance varies across the finance providers and the measures of risk. For instance, finance obtained as commercial credit from banks was associated with a higher 25^th^ percentile, implying a lower production risk in terms of the absolute yield level. This is probably because commercial credit from banks is often for large loans that can be used to improve irrigation to increase production [20]. At the same time however, commercial credit was negatively associated with the skewness of the yield distribution, indicating that the yield distribution becomes relatively more left-skewed. The negative association with the skewness of the yield distribution suggests that the access to finance does not preclude very low yields. For instance, irrigation may help farmers to increase their production, but it does not preclude flooding [54], and hence does not preclude extremely low yields as a result of flooding. The negative association with skewness was also found for finance obtained from farmers’ associations. This association might arise if the in-kind finance is not used to benefit the crops. For instance, Gow & Swinnen [59] found that in-kind finance was used for other purposes, such as selling the inputs, and therefore did not benefit overall farm production. The negative association with skewness was also might arise because of the issue of trust as Baranyai et al. [60] and Vasa et al. [61] revealed the importance of faith in loyalty affecting farmers’ cooperation activity.

**Table 3:** Coefficients from the ordinary least square’s regression of production risk on the explanatory factors (standard errors in parentheses).

Variables		Coefficient of variation	Skewness	Kurtosis	25^th^ percentile*
**Bank**	**Commercial credit**	-0.03 (0.02)	-0.24 ^c^ (0.13)	0.37 (0.36)	0.06 ^c^ (3.45)
	**Subsidized credit**	-0.02 (0.02)	0.03 (0.12)	-0.27 (0.28)	0.04 (3.68)
**MFI**		-0.01 (0.03)	0.03 (0.16)	0.39 (0.26)	0.04 (3.47)
**Farmers’ association**		0.00 (0.02)	-0.13 ^c^ (0.07)	-0.15 (0.14)	0.03 (2.38)
**Trader**		-0.01 (0.02)	-0.12 (0.10)	0.44 (0.29)	-0.00 (3.21)
**Kiosk**		-0.00 (0.02)	0.17 ^c^ (0.09)	-0.37 ^b^ (0.19)	-0.05 ^c^ (2.86)
**Others**		0.00 (0.01)	0.05 (0.06)	-0.30 ^b^ (0.13)	-0.03 (2.17)
**Age**		0.00 (0.01)	-0.04 (0.05)	0.08 (0.13)	-0.00 (1.45)
**Education**		-0.01 (0.01)	0.04 (0.03)	-0.07 (0.07)	-0.01 (1.03)
**Farming experience**		0.01 (0.01)	0.02 (0.05)	0.10 (0.12)	0.00 (1.47)
**Farm size**		0.01 (0.01)	-0.04 (0.04)	0.40 (0.05)	0.02 ^c^ (1.28)
**Distance to kiosk**		0.02 (0.01)	0.05 (0.03)	0.02 (0.08)	-0.03 ^c^ (1.10)
**Infrastructure**		-0.01 (0.02)	0.14 ^c^ (0.08)	0.01 (0.19)	-0.02 (2.20)
**Mangosteen**		0.01 (0.03)	-0.01 (0.09)	-0.04 (0.17)	0.05 (2.92)
**Chili**		0.00 (0.02)	-0.04 (0.10)	0.68 ^a^ (0.23)	0.06 ^c^ (3.16)
**Red Onion**		-0.05 ^a^ (0.02)	-0.31 ^c^ (0.10)	0.35 ^b^ (0.16)	0.10 ^b^ (3.24)
**Constant**		0.26 (0.02)	-0.07 (0.08)	2.30 (0.13)	0.23 (2.31)

^a^ Significant at 1%,

^b^ 5%, and

^c^ 10% level.

* The unit was rescaled on a scale from 0 to 1 by subtracting the minimum value from each yield value and dividing the result by the difference between the maximum and minimum values.

Source: Authors’ own calculation.

Farmers with access to finance from the agricultural input kiosk had a lower 25^th^ percentile as well as the kurtosis, implying a lower absolute level of yield and a flatter distribution. This may reflect that farmers who access finance from kiosks are generally those having lower yields; Wulandari et al. [28] found that mainly less-educated farmers use this source of finance. At the same time, farmers with access to finance from the kiosk had a larger skewness, i.e. the yield distribution becomes relatively less left-skewed. This suggests that very low yields are less likely to occur for these farmers, probably because they have direct access to pesticides. In line with the results for finance obtained from input kiosks, finance from other sources also flattened the yield distribution, as indicated by the lower kurtosis. A possible explanation might be that the farmers applying for other sources are the ones with less experience and therefore they may not be able to fully benefit from the obtained finance.

With respect to socio-economic factors, significant associations were only found for farm size, distance to agricultural input kiosk and road infrastructure. Farm size positively associated with the 25^th^ percentile, implying that larger farms are less likely to face lower absolute yield levels per hectare. This is probably because larger farmers can use crop diversification to increase the likelihood of adapting to climate change [52]. Distance to kiosk negatively associated with the 25^th^ percentile, implying that larger distances to a kiosk potentially lead to higher production risk, which is consistent with our prior expectation. Infrastructure was positively associated with the skewness of the yield distribution, which suggests that having better roads leads to less production risk in terms of the skewness of yields. Studies have found that good roads increase access to social services, such as farm inputs and credit markets [54, 55], which may explain our finding that good road infrastructure lowers the skewness measure of production risk.

The results for the farm specialization dummy variable show that, compared to the other crops, red onion farms have the lowest production risk in terms of CV and 25^th^ percentile (Table 4). Nevertheless, the negative skewness still illustrates their high vulnerability for extreme weather events. Adiyoga [62] showed that crop farmers in Indonesia, especially red onion farmers, experienced a decline in yield growth during the period from 1969 to 2006, probably related to climate change [63].

**Table 4:** Marginal effects from the ordinary least square’s regression of production risk on the explanatory factors (standard errors in parentheses).

Variables	Coefficient of variation	Skewness	Kurtosis	25^th^ percentile*
**Mango**	0.25 ^a^ (0.01)	-0.04 (0.05)	2.22 ^a^ (0.07)	0.22 ^a^ (1.69)
**Mangosteen**	0.26 ^a^ (0.03)	-0.05 (0.07)	2.18 ^a^ (0.15)	0.27 ^a^ (2.48)
**Chili**	0.25 ^a^ (0.02)	-0.09 (0.08)	2.91 ^a^ (0.23)	0.28 ^a^ (2.43)
**Red Onion**	0.20 ^a^ (0.01)	-0.35 ^a^ (0.08)	2.57 ^a^ (0.14)	0.32 ^a^ (2.68)

^a^ Significant at 1% level.

* The unit was rescaled on a scale from 0 to 1 by subtracting the minimum value from each yield value and dividing the result by the difference between the maximum and minimum values.

Source: Authors’ own calculation.

## Conclusions

This study analyzed the association between the perceived production risk of horticultural farms and access to finance from different finance providers, such as commercial and subsidized credit from banks, commercial credit from MFI, in-kind finance provided by farmers’ associations, in-kind finance from traders, flexible payments of inputs to agricultural input kiosks and other finance sources. Data were collected from 434 Indonesian horticultural farmers who cultivate mango, mangosteen, chili, and red onion. Production risk was measured by the CV, skewness, kurtosis and the 25^th^ percentile of the elicited yield distributions.

The results show that the association between production risk and access to finance varies across the finance providers. There is no finance provider for which we found only risk-lower association. Depending on the risk measure, some risk-lower associations were found for commercial credit from banks and flexible payments of inputs to kiosks. We found no risk-lower associations for in-kind finance from farmers’ associations and finance from other sources. With regard to socio-economic factors, we found that larger farm size, close proximity to an agricultural input kiosk and good road infrastructure are associated with lower production risk.

The results of this study provide insights to policy makers and finance providers about the association between different sources of finance and lower production risk. The findings suggest that although access to finance does appear to have a lower production risk, this association is complex and dependent on who provides the finance and under which conditions. From our findings, we recommend that public and private initiatives to provide finance to farmers should prioritize commercial credit programs from banks and flexible payments of inputs to agricultural input kiosks.

## Supporting information

S1 Appendix (DOCX)

S1 File Questionnaire for farmers. (DOCX)

S1 Table Data of farmers. (XLSX)
