# Supplementary material for: The role of access to finance from different finance providers in production risks of horticulture in Indonesia
Source: PLoS One. 2021 Sep 27;16(9):e0257812. doi: 10.1371/journal.pone.0257812 (PMC8475986; doi:10.1371/journal.pone.0257812)
Supplement: S1 Appendix — (DOCX) [file pone.0257812.s001.docx]

S1 Appendix.

**Table A. Descriptive statistics of perceived yields**

| **Variables** | **Mango** | **Mangosteen** | **Chili** | **Red Onion** |
| --- | --- | --- | --- | --- |
| **Minimum** | 103.43 | 21.08 | 10.48 | 6.40 |
| **Maximum** | 273.41 | 59.90 | 23.87 | 11.94 |
| **Mean** | 175.99 | 37.94 | 17.74 | 9.59 |
| **Standard deviation** | 52.00 | 12.63 | 4.27 | 1.85 |

The statistic is calculated as the mean and the value is in 1000 kilograms

Source: authors’ own calculation
