# Supplementary material for: The role of access to finance from different finance providers in production risks of horticulture in Indonesia
Source: PLoS One. 2021 Sep 27;16(9):e0257812. doi: 10.1371/journal.pone.0257812 (PMC8475986; doi:10.1371/journal.pone.0257812)
Supplement: S1 File — (DOCX) [file pone.0257812.s002.docx]

**S1 File. Questionnaire for farmers**

**The role of access to finance from different finance providers in production risks of horticulture in Indonesia**

Farmer ID

| Name of Farmer |  |
| --- | --- |
| Address |  |
| Interviewer |  |

1. Farmer and farm characteristics

| 1.1. Age (years) |  |
| --- | --- |
| 1.2. Educational background (years) |  |
| 1.3. Experience in agricultural activities (years) |  |
| 1.4. Total farm size (hectares) |  |
| 1.5. Distance to kiosk (kilometers) |  |
| 1.6. Infrastructure (1: good roads; 0: no good roads) |  |

1. Access to finance

| 2.1. Did you obtain one of the following sources of finance in 2013? | Yes | No |
| --- | --- | --- |
| 1. Commercial credit from bank |  |  |
| b. Subsidised credit from bank |  |  |
| c. Commercial credit from microfinance institution |  |  |
| d. In-kind finance from farmers’ association |  |  |
| e. In-kind finance from trader |  |  |
| f. Flexible payment to agricultural input kiosk |  |  |
| g Finance from family/relatives/neighbour/friends |  |  |

- 1. From twenty coins given to you, how many coins would you distribute to the following columns showing expected yield that may obtain for the next five years? The expectation can be adjusted regarding previous yield, farm condition and farmers’ capacity in farming. The twenty coins were given to the farmers are representing a 0.05 probability.

|  | Minimum |  |  |  |  |  | Maximum |
| --- | --- | --- | --- | --- | --- | --- | --- |
|  | 1 | 2 | 3 | 4 | 5 | 6 | 7 |
| Yield |  |  |  |  |  |  |  |
| Number of coins |  |  |  |  |  |  |  |

Thank you very much for your participation.
